# A congenital case of meningomyelocele in a newborn

**DOI:** 10.11604/pamj.2025.52.155.48239

**Published:** 2025-12-11

**Authors:** Pranjali Saranjami Jode, Deeplata Mendhe

**Affiliations:** 1 Community Health Nursing Department, Smt. Radhikabai Meghe Memorial College of Nursing, Sawangi (Meghe), Wardha, Maharashtra, India

**Keywords:** Meningomyelocele, spina bifida, neural tube defect, lumbosacral region

## Image in medicine

A two-day-old male infant was brought to the neonatal unit due to a soft, fluid-filled swelling in the lower back present since birth. The swelling was located in the lumbosacral region and was observed immediately post-delivery. The infant had not urinated since birth and showed no movement in the lower limbs. The baby, born via an uncomplicated vaginal delivery weighing 3.2 kg, had no perinatal complications. The mother had received all recommended prenatal immunisations and routine antenatal care. On examination, a sac-like mass covered by thin, translucent skin with visible neural tissue was noted in the lower back. This presentation was consistent with meningomyelocele, the most severe form of spina bifida. It results from the failure of neural tube closure during the third to fourth week of embryonic development, leading to herniation of the spinal cord and meninges through a vertebral defect. Meningomyelocele is among the most common congenital central nervous system anomalies. Risk factors include folic acid deficiency, genetic predisposition, maternal diabetes, and teratogenic medications. Diagnosis is often made prenatally via ultrasound or postnatally through physical examination. To prevent infection of the exposed neural tissue, prophylactic antibiotics were administered. A neurosurgical consultation was obtained, and urgent surgical repair was planned. Long-term management includes monitoring for hydrocephalus, orthopaedic deformities, and neurogenic bladder. Multidisciplinary follow-up and rehabilitation are essential to optimise neurological function and quality of life in affected infants. Early intervention is critical to improving outcomes and supporting development.

**Figure 1:**
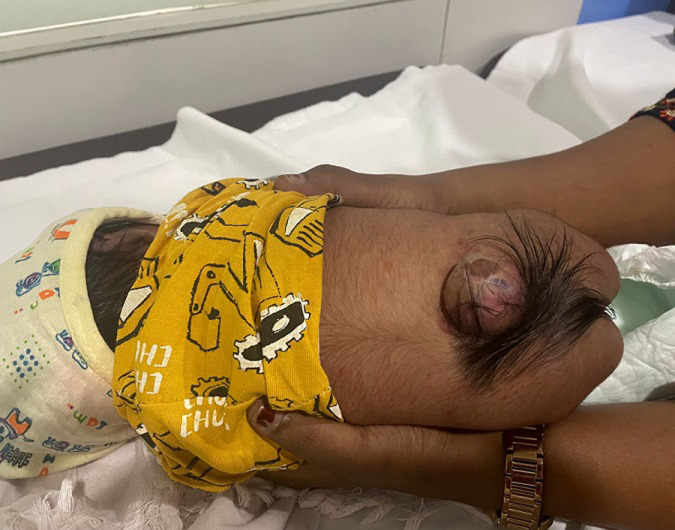
meningomyelocele in the lumbosacral region

